# Supplementary material for: Leveraging Attention-Based Convolutional Neural Networks for Meningioma Classification in Computational Histopathology
Source: Cancers (Basel). 2023 Oct 28;15(21):5190. doi: 10.3390/cancers15215190 (PMC10647687; doi:10.3390/cancers15215190)
Supplement: Supplementary file 1 [file cancers-15-05190-s001.zip › cancers-2638587-supplementary.pdf]

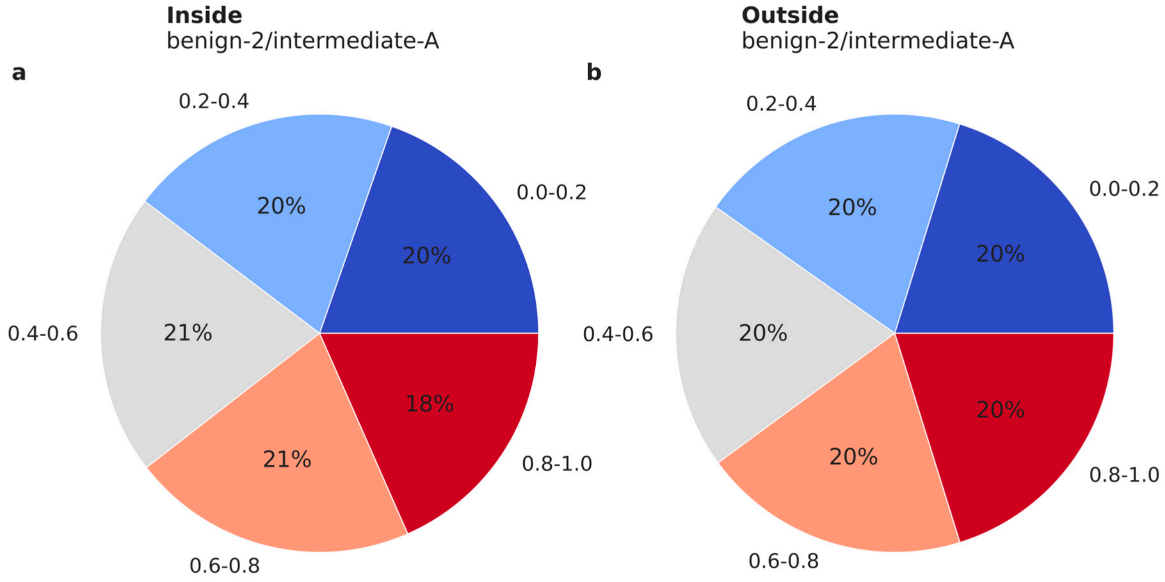

Figure S1: Attention map statistics of benign-2 vs. intermediate-A classification. (a,b) Attention map statistics to evaluate the relative proportion of high and low attention values inside (a) and outside (b) the tumor areas, highlighted by a neuropathologist. The color grades were grouped into 5 discrete classes. Only attention maps at 20_ were considered for this evaluation, and proportion was computed per attention map and averaged overall. Methylation classes are benign-2 (n = 18) and intermediate-A (n = 17).

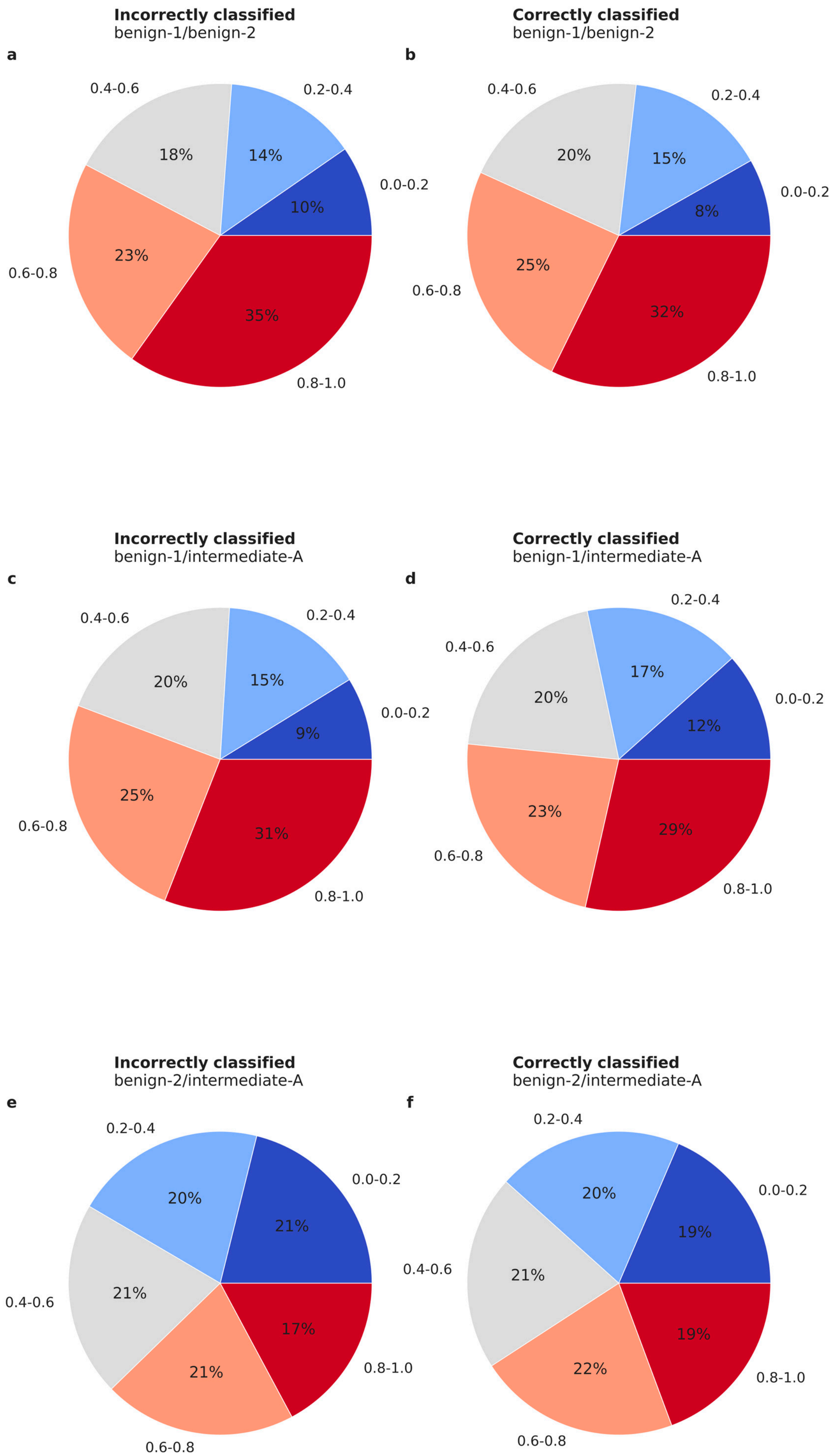
Incorrectly classified
benign-1/benign-2
a
0.4-0.6
0.2-0.4
18%
14%
10%
0.0-0.2
23%
0.6-0.8
35%
0.8-1.0
Correctly classified
benign-1/benign-2
b
0.4-0.6
0.2-0.4
20%
15%
8%
0.0-0.2
25%
0.6-0.8
32%
0.8-1.0
Incorrectly classified
benign-1/intermediate-A
c
0.4-0.6
0.2-0.4
20%
15%
9%
0.0-0.2
25%
0.6-0.8
31%
0.8-1.0
Correctly classified
benign-1/intermediate-A
d
0.2-0.4
0.4-0.6
17%
20%
12%
0.0-0.2
23%
29%
0.6-0.8
0.8-1.0
Incorrectly classified
benign-2/intermediate-A
e
0.2-0.4
0.0-0.2
20%
21%
0.4-0.6
21%
17%
21%
0.8-1.0
0.6-0.8
Correctly classified
benign-2/intermediate-A
f
0.2-0.4
0.0-0.2
20%
19%
0.4-0.6
21%
19%
22%
0.8-1.0
0.6-0.8

Figure S2: Attention map statistics of all three classification tasks to evaluate the relative proportion of high and low attention values inside the tumor areas. (a,b) Attention map statistics of benign-1 vs. benign-2, with (a) considering only incorrectly classified samples and (b) considering only correctly classified. (c,d) Attention map statistics of benign-1 vs. intermediate-A, with (c) considering only incorrectly classified samples and (d) considering only correctly classified. (e,f) Attention map statistics of benign-2 vs. intermediate-A, with (e) considering only incorrectly classified samples and (f) considering only correctly classified. The color grades were grouped into five discrete classes. Only attention maps at 20_ were considered for this evaluation, and proportion was computed per attention map of incorrectly classified samples (a,c,e), respectively of correctly classified samples (b,d,f), and averaged over all samples. Methylation classes are benign-1 (n = 13), benign-2 (n = 18), and intermediate-A (n = 17).
